# The COSMIC Bubble Helmet: A Non-Invasive Positive Pressure Ventilation System for COVID-19

**DOI:** 10.1109/OJEMB.2020.3036742

**Published:** 2020-11-09

**Authors:** Vionarica Gusti, Wan Jun Wu, Arpan Grover, Sabian Chiu, Kai-Wen Su, Erica Ma, Chanelle K. Chow, Ella Sit, Jun Lim, Abhijit Pandhari, Mattias Park, Ryan Lee, Faisal Shahril, Shawn T. Lim, Christopher Y. Nguan, Dan Driedger, Avinash K. Sinha, Ivan G. Scrooby, Neilson J. Mclean, Michael W. Lee, Tyler D. Yan, The COSMIC Team

**Affiliations:** Faculty of MedicineUniversity of British Columbia 8166 BC V1Y 1T3 Canada; Department of Integrated Engineering, Faculty of Applied ScienceUniversity of British Columbia 8166 Vancouver BC V6T 1Z4 Canada; Department of Biomedical Engineering, Faculty of Applied ScienceUniversity of British Columbia 8166 Vancouver BC V6T 1Z4 Canada; Institute of Biomedical Engineering, National Taiwan UniversityOrthopedic Engineering and Motion Analysis Laboratory 33561 Taipei City 10617 Taipei; Department of BiologyUniversity of British Columbia 8166 Vancouver BC V6T 1Z4 Canada; Department of Material Engineering, Faculty of Applied ScienceUniversity of British Columbia 8166 Vancouver BC V6T 1Z4 Canada; Faculty of Engineering and Computer Science, Department of Computer ScienceUniversity of Victoria 8205 Vancouver BC V8P 5C2 Canada; Bot Camp Toronto M3B 2R7 Canada; Department of Urological SciencesUniversity of British Columbia 8166 Vancouver BC V5Z 1M9 Canada; Vancouver General HospitalBiomedical Engineering 199005 Vancouver BC V5Z 1M9 Canada; Department of MedicineMcGill University 5620 Montreal QC H4A 3J1 Canada; Cariboo Memorial Hospital 103261 Williams Lake BC V2G 2G8 Canada; Abbotsford Regional Hospital and Cancer Centre 60448 Fraser Health Authority Abbotsford BC V2S 0C2 Canada; The COSMIC Team Vancouver BC Canada

**Keywords:** COVID, helmet, non-invasive, pressure, ventilator

## Abstract

*Goal:* COSMIC Medical, a Vancouver-based open-source volunteer initiative, has designed an accessible, affordable, and aerosol-confining non-invasive positive-pressure ventilator (NIPPV) device, known as the COSMIC Bubble Helmet (CBH). This device is intended for COVID-19 patients with mild-to-moderate acute respiratory distress syndrome. *System Design*: CBH is composed of thermoplastic polyurethane, which creates a flexible neck seal and transparent hood. This device can be connected to wall oxygen, NIPPVs including Continuous Positive Airway Pressure and Bi-level Positive Airway Pressure, and mechanical ventilators. *Discussion*: Justification of CBH design components relied on several factors, predominantly the safety and comfort of patients and healthcare providers. *Conclusion:* CBH has implications within and outside of the pandemic, as an alternative to invasive mechanical ventilation methods. We have experimentally verified that CBH is effective in minimizing aerosolization risks and performs at specified clinical requirements.

## I. Introduction

As COVID-19 continues to spread globally, the combined threat to patients that have developed acute respiratory distress disorder (ARDS), and the potential for airborne transmission of highly contagious SAR-CoV-2 coronavirus remains a central concern among the medical community [1]. The standard of care treatment for COVID-19 related ARDS includes both invasive mechanical ventilation and non-invasive positive-pressure ventilation (NIPPV) [2]. In patients suffering from milder cases of hypoxemia [3] or ARDS [4], NIPPV has been proposed as the optimal method of oxygenation to stabilize this subset of patients [3]–[5]. Locally, of the 117 COVID-19 patients admitted to the ICU in Vancouver, British Columbia, 12.8% of these patients were treated using NIPPV [6]. Other studies have indicated that the percentage of COVID-19 patients placed on NIPPV treatment varies between 13%–56% [7], [8]. These values, if not more, may represent the proportion of patients that may avoid invasive ventilation because of devices like CBH. These values emphasize the possible benefits of NIPPV treatment as invasive ventilation methods may contribute to ventilator-induced lung injury in fragile lung tissues [9], therefore amplifying the risks associated with mechanical ventilation.

The *European Society of Intensive Medicine's guidelines for COVID-19 patient management* currently do not recommend conventional NIPPV due to virus aerosolization risks to healthcare employees and others in the environment [9]. This has limited NIPPV administration to isolation rooms using negative pressure systems, and in many cases, premature escalation of invasive ventilation [10] when a patient's respiratory demand exceeds conventional high-flow oxygen masks. However, helmet-based NIPPVs have been shown to reduce intubation and mortality rates by upwards of 43% and 22%, respectively, compared to traditional face masks, and are a possible solution for minimizing NIPPV-associated aerosolization risks [11]. Helmet designs are better tolerated by patients due to increased comfort compared to traditional NIPPV modalities, and provide the patient with clearer, unobstructed views compared to full-face mask designs [12].

COSMIC Medical, a Vancouver-based non-profit organization, offers a non-rigid ring helmet-based ventilatory system design that is open-source, affordable, and accessible, known as the COSMIC Bubble Helmet (CBH). While the CBH is specifically designed for COVID-19 patients, this device provides a potential NIPPV solution in many clinical scenarios where pre-ventilation respiratory support is required. CBH may also play a role in optimizing critical care resources by obviating the need for invasive ventilation in some cases, freeing ICU equipment for patients in fulminant respiratory distress.

## II. System Design

**Fig. 1.**
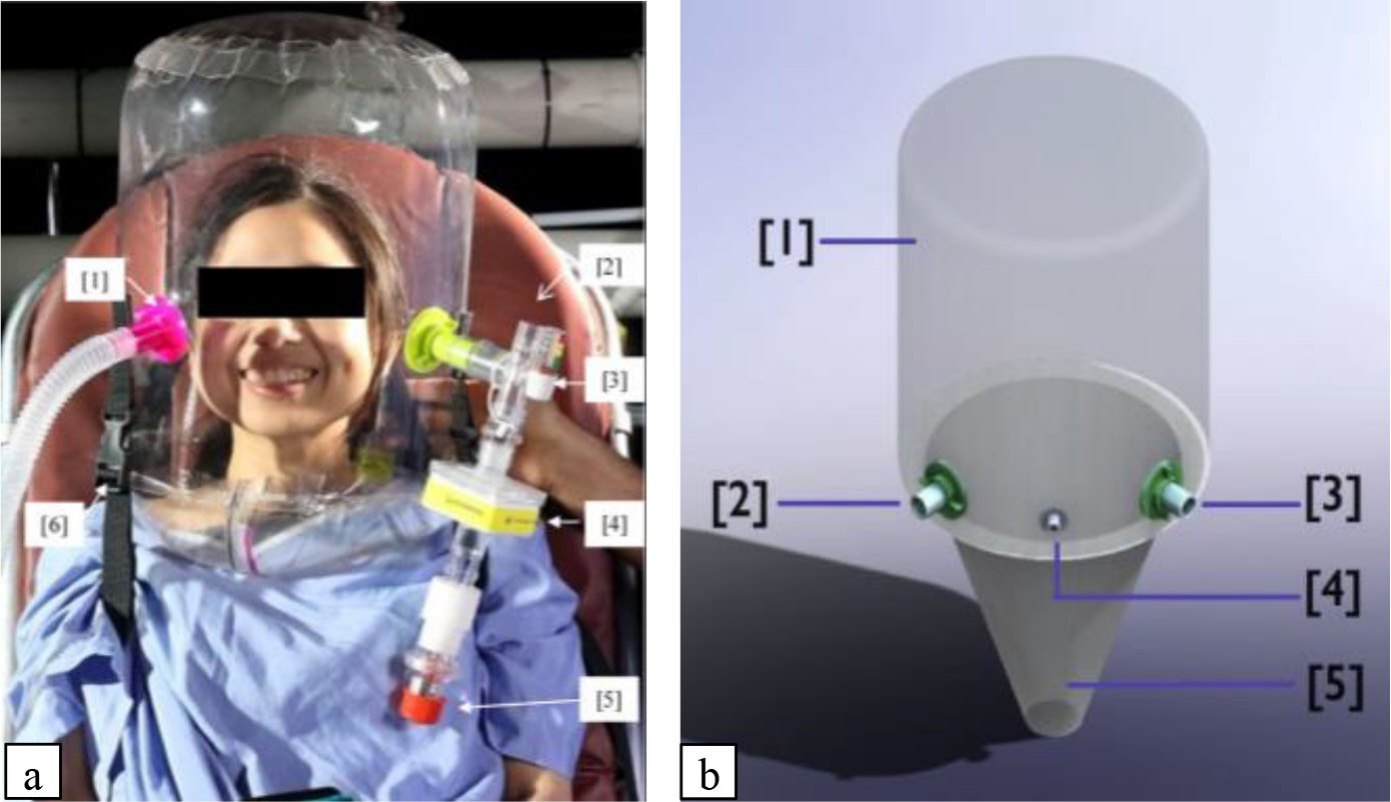
(a) COSMIC bubble helmet connected to wall oxygen - air supply via inspiratory ports, 3D printed 22mmOD ports [1]. Expiratory port gas exhaust assembly, which consists of a T-piece with one-way valve and manometer connection [2], a manometer [3], a high-efficiency particulate air (HEPA) filter [4], and a positive end-expiratory pressure (PEEP) valve [5], adjustable strap buckle [6]. (b) Render of the COSMIC Bubble Helmet. Clear hood [1], inspiratory port [2], expiratory port [3], feeding tube access port (optional) [4], neck gasket [5].

**Fig. 2.**
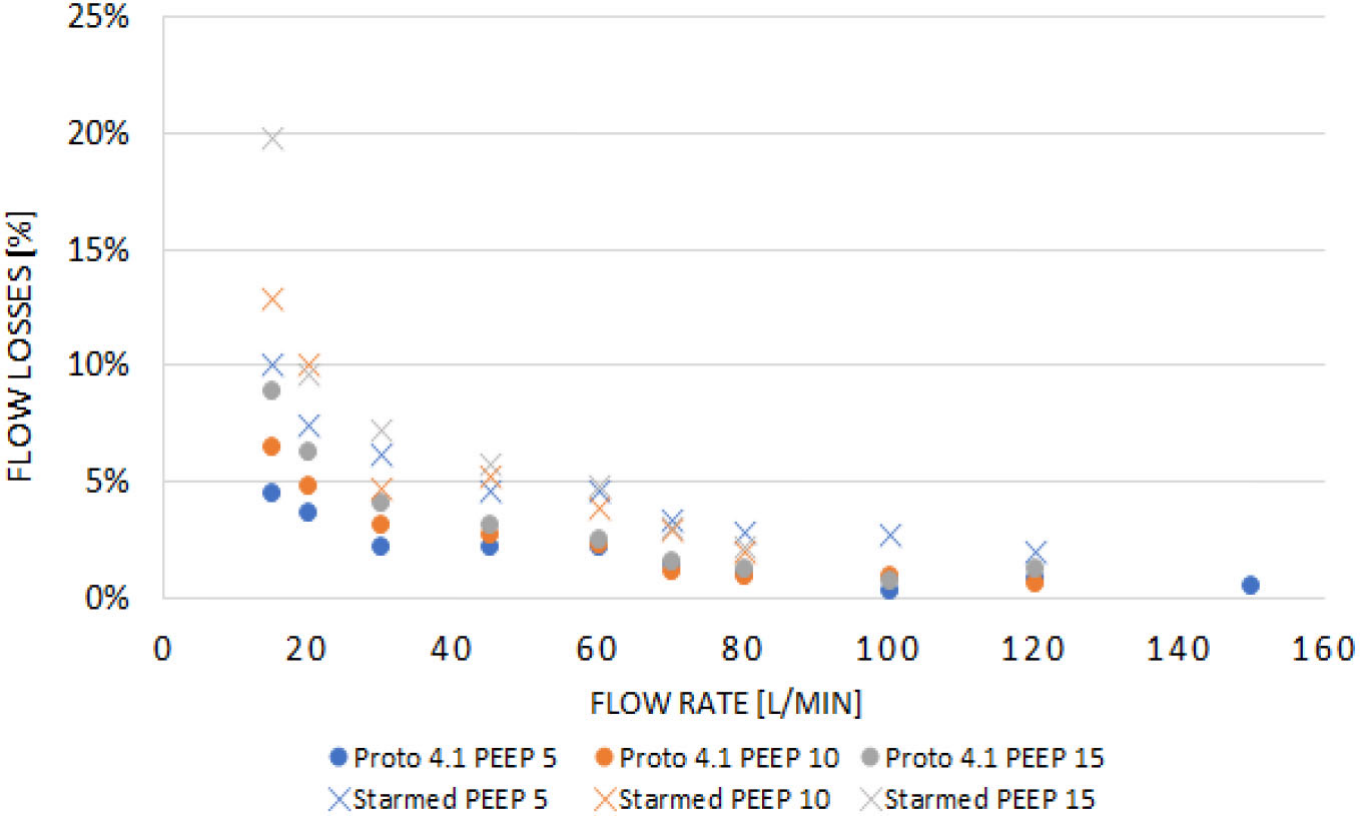
Performance of the CBH (−) vs CaStar CPAP hood 210M helmet with an occluded manometer air vent (—) in terms of neck seal leakage in glass mannequin head. Leakage measured as percentage difference of flow rate measured at inflow and outflow port, Flow Losses (%) vs Flow Rate (L/min) in varying PEEP level 5 cmH_2_O ($\boldsymbol\bullet$), 10cmH_2_O (▲), 15cmH_2_O (■).

Due to the lack of well-established published standards for helmet respiratory interfaces, we based our design on existing helmets' published literature, considering the functionality, safety, feedback from healthcare practitioners, and ease of device production. The CBH (Fig. 1) consists of two major components: a flexible, transparent hood, and a neck-seal. The CBH hood and neck seal are composed of biocompatible thermoplastic polyurethane (TPU). The current thickness of the CBH TPU is 0.381 mm for the hood and 0.076 mm for the neck seal (polyether, Shore 82A). These two different thicknesses allow for compatibility with heat welding techniques, such as impulse sealing or radio-frequency welding. These heat welding techniques attach the neck seal and the hood to a non-rigid ring. The in-folding of the conically shaped neck seal provides a hermetic seal when the helmet is pressurized, while also minimizing patient neck restriction. The thinner gauge TPU conforms to the patient's neck contour and provides a better seal at a higher flow rate, less than 3% leakage at 40L/mins and above (Fig. 2). It also provides minimal resistance during patient donning, allowing the neck seal to be stretched over the patient's head, all while maintaining the seal and minimizing leakage when pressurized.

Table I summarizes our specifications that CBH has achieved. The non-rigid ring design of CBH provides greater freedom of mobility as patients can comfortably lie supine or prone without affecting the effectiveness or the performance of the neck seal and/or hood. Shoulder straps with quick-release buckles ensure that the device is properly secured when in use, yet easily removed in the case of an emergency. Adjusting the length of shoulder straps allows for the adjustment of the helmet height to optimize helmet volume, thus minimizing CO_2_ rebreathing. The neck seal will first be cut to an opening diameter that is smaller than the patient's neck circumference to minimize aerosol leakage. To achieve this, the neck seal was cut at 40% of the patient's neck circumference; however, an optimal neck seal opening must be determined through further testing. The two sets of 22mm outer diameter ports in the hood's body allow the device to be used with hospital wall oxygen, Continuous Positive Airway Pressure (CPAP), or Bi-Level Positive Airway Pressure (BiPAP) devices, or ventilators. When connected to a wall oxygen air supply, the amount of respiratory support for patient airways is controlled by varying the resistance provided by the PEEP valve and the airflow rate. The amount of pressure support can be adjusted by CPAP, BiPAP, or ventilator machines independently. A HEPA filter is used to contain the virulent aerosolized particles released by the patient, ensuring clean output airflow from the CBH system to protect healthcare workers and bystanders.

**TABLE I:** Product Specifications for the COSMIC Bubble Helmet

ID	Specification
**Technical Specifications (TS)**	
**TS1**	Utilizes biocompatible thermoplastic polyurethane, compatible with high oxygen concentration, and has minimal outgassing
**TS2**	Able to withstand an airflow of up to 150 L/min and positive end-expiratory pressure (PEEP) of up to 30 cmH_2_O.
**TS3**	Has air leakage below 3% at flow rate greater than 40L/min.
**Usability Specifications (US)**	
**US1**	Has a cut-to-size neck seal that is based on the patient's neck size.
**US2**	Helmet donning can be performed in less than one (1) minute.
**US3**	The transparency of the helmet allows the provider to observe patients clearly through the device, minimizes feelings of claustrophobia and facilitates communication.
**US4**	Allows patients to speak and cough without hindrance and with less chance of asphyxiation.
**US5**	The noise within the helmet does not exceed 87dB for 8 hours as per Canadian Federal Noise Regulation.
**US6**	The non-rigid ring that acts as an attachment point between the neck seal and helmet allows for minimal discomfort in a prone position, side position and in an upright position while wearing the device. The neck seal should offer flexibility in terms of patient movement and positioning.
**US7**	The CBH has shoulder straps that are easily removable.
**Safety Specifications (SS)**	
**SS1**	The CBH can be easily removed by the patient or health care workers. In the case of an emergency, a pair of scissors can be used to cut open the CBH
**SS2**	The CBH requires a minimum flow of 30 L/min to prevent inspired CO_2_ accumulation greater than 7.6 torr as per ISO 17510
**Market Specifications (MS)**	
**MS1**	The cost of CBH is aimed to be under $100 CAD once mass production begin
**MS2**	Utilizes materials that are available in local industry and has a lower initial investment manufacturing cost

## III. Discussion

The CBH arose from our desire to offer a novel noninvasive respiratory support device for the management of COVID-19 patients. The initial prototype was inspired by the AMRON hyperbaric oxygen treatment helmet, which has a rigid ring mechanically attaching the elastic neck seal to the transparent hood. Rigid ring designs pose challenges in prototyping costs, 3D printer usage, complex injection molding design requirements, and neck seal mold casting. Most importantly, rigid rings impact patient comfort while in a prone position, which is considered to be essential for increasing oxygenation and reducing mortality rates in patients with severe ARDS [13], [14].

Transitioning to a single-material design allowed the development of a smooth transition between the hood and neck seal. We selected TPU as our material of choice for its elastic properties, thermal weldability, optical clarity, minimal outgassing, and biocompatibility [15]. TPU films can be used for both the hood and the neck seal at varying thicknesses, allowing for a simplified, single-material design. As a result, this simplified design requires a low initial investment and minimal retooling by local manufacturers with plastic welding capabilities. The simplicity of CBH design maintains the core functionality of NIPPV and aerosol containment while offering increased patient comfort. Future considerations, such as the addition of a feeding port, air-tight zipper, pull tab for emergency doffing, and larger anti-asphyxiation valve, may be considered. Moreover, manufacturing of CBH can be performed efficiently if a rig is created solely for device production. In our development effort, we conducted several benchmark tests with CaStar CPAP hood, which shows comparable performance in terms of leakage, pressure and airflow relationship, noise level, and CO_2_ rebreathing (Supplementary Materials section III). The results of the testing showed comparable data to published results of another commercial helmet NIPPV.

Before offering CBH to patients, there remain steps to be taken including obtaining regulatory approval, and continuing diligence regarding safety, efficacy, design iteration and clinical trials. We will also need to expand testing for other benchmarks, including BiPAP generation, and research means of determining airflow losses within clinical settings. We will also need to create methods for clinicians and other healthcare professionals to effectively measure and minimize air leakage outside of experimental conditions. To determine the CBH's efficacy in ventilating patients who have compromised respiratory control, clinical studies need to be performed with a larger patient sample size.

This currently open source COSMIC design is available on GitHub and will facilitate innovation of this simple and effective concept of helmet NIPPV. Please refer to Section I of the Supplementary Materials for the link to CBH's main repository.

## IV. Conclusion

The CBH is a NIPPV system designed as a low-cost treatment method that can be used in emergency situations to support patients in respiratory distress from COVID-19 and other disease states, as well as help to mitigate ventilator shortages in low resource environments. Major design considerations were given to adequate respiratory support, aerosol containment, patient positioning for ARDS, and an easily reproducible, affordable design. CBH has undergone usability testing and has been shown to perform equivalently to the CaStar CPAP hood in minimizing aerosolization risks and meeting the specified clinical treatment requirements. In future, we intend to test for the device's ability to produce bi-level positive airway pressure in order to determine the its full efficacy within clinical settings.

## Supplementary Materials

Please see the online supplementary information. There you will find more information about our research group, our GitHub repository and functionality and usability testing data.


