# Supplementary material for: The COSMIC Bubble Helmet: A Non-Invasive Positive Pressure Ventilation System for COVID-19
Source: IEEE Open J Eng Med Biol. 2020 Nov 9;1:312–5. doi: 10.1109/OJEMB.2020.3036742 (PMC8545034; doi:10.1109/OJEMB.2020.3036742)
Supplement: Supplementary file 1 [file supp1-3036742.pdf]

Supplementary Materials

The COSMIC Bubble Helmet: A Non-Invasive Positive Pressure Ventilation System for COVID-19

Vionarica Gusti, Wan Jun Wu, Arpan Grover, Sabian Chiu, Kai-Wen Su, Erica Ma, Chanelle K. Chow, Ella Sit, Jun Lim, Abhijit Pandhari, Mattias Park, Ryan Lee, Faisal Shahril, Shawn T. Lim, Christopher Y. Nguan, Dan Driedger, Avinash K. Sinha, Ivan G. Scrooby, Neilson J. Mclean, Michael W. Lee, Tyler D. Yan and COSMIC

I. Our Group

OUR organization, the Collective Open-Source Medical Innovations for COVID-19 (COSMIC), is a Vancouver, British Columbia-based non-profit composed of over 130 students, physicians, engineers, and other professionals in a variety of fields. The organization's mission is to provide respiratory support devices and personal protective equipment (PPE) designs via open source to a global audience. Our open sourced design can be found at: https://github.com/COSMIC-medical/bubble-helmet

The promise of helmet based NIPPV has led to the development of the COSMIC Bubble Helmet NIPPV. As a subset of COSMIC Medical, our Project Bubble Helmet team has collaborated on the design, technical writing, and manufacturing of this adapted device. While this design aims to tackle virus aerosolization and ventilatory demands in patients with ARDS due to COVID-19, CBH has applications in patients with respiratory distress of any etiology.

II. Requirements

The requirements were defined through literature review, consultation with key stakeholders including doctors and respiratory therapists. Assumptions in the benchmarking tests of the bubble helmet are detailed below.

A. CO_2 Accumulation Justification

A concern for helmet-based ventilation is the potential for CO_2 accumulation and rebreathing in the helmet. Taccone's study on the effects of helmet ventilation on carbon dioxide rebreathing was carried out in healthy volunteers (n=8) and provided evidence that CO_2 levels are not related to the applied PEEP or hood volume [16]. Taccone's research builds upon previous studies demonstrating that the hood's CO2 concentration is independent of the PEEP and depends on the gas flow [17]. This substantiates the assumption that CO_2 accumulation is dependent on flow and thus tested in detail for the CBH (below).

B. Operational Noise Level Justification

Another problem for helmet delivery of CPAP with the helmet is the noise level for the patient. A recent study of two alternative NIV helmets, the StarMed and Dimar hoods, reported similar performances in noise levels at a gas flow of 40 L/min compared to 20 L/min (74 dBA vs. 52 dBA respectively) [18]. The authors also suggest that a diffuser filter should not be used with gas flows over 30 L/min because it will amplify the sound pressure.

III. Functionality Testing Data

The CBH has been tested iteratively throughout its development to inform its design and ensure its efficacy. These tests included benchmark testing to evaluate its performance, ensure safe airflow characteristics, and usability testing to assess its suitability for use by healthcare providers.

A. Leakage Test

The purpose of the leakage test was to measure the losses in airflow due to leaks in the helmet by quantifying air dispersion levels. Measuring air dispersion allows a quantitative measure of the helmet's structural integrity and its ability to control aerosolized viral spread under operating conditions. Air dispersion should be minimized to avoid contamination of the environment. By observing the difference between inflow and outflow, the rate of leakage can be determined. The leakage was compared to that of the StarMed CPAP Hood, which is a Health Canada and FDA-cleared equivalent (Figure 2).

The CBH registers consistently lower air leakage than the StarMed hood at PEEP valve settings of 5, 10, and 15 cmH_2O. In a recent study performed by Feriolin, the StarMed helmet with air cushion had a negligible air dispersion radius compared to the one-meter air dispersion radius of high-flow nasal cannula [19]. The leakage testing performed provides evidence that the CBH has lower rates of air leakage compared to CaStar StarMed helmet and can reduce virus aerosolization from air dispersion.

B. CO_2 Accumulation Test

The CO_2 accumulation test evaluates the accumulation of CO_2 within the CBH when in use. With a healthy test subject and a CO_2 monitoring probe, the helmet was tested with a range of flow rates to determine the flow necessary to prevent dangerous CO_2 rebreathing.

We demonstrated that with two human subjects, one male and one female, a minimum flow rate of 30L/min is required to prevent an increase in $FiCO_2$ above 7.6mmHg based on the ISO 17510 (Figure S1). The subjects in these experiments were healthy young adults in a sitting resting position. The recommended level for therapeutic patient use will be determined in a future clinical trial feasibility study of the CBH and beyond the scope of this experiment. We demonstrated that in our small sample, the CBH is safe and comfortable to use for the duration of the experiment (approx. 60 minutes). Subjects

reported no clinical signs of CO_2 rebreathing during the experiment. Future experiments will be conducted to evaluate CO2 accumulation in prolonged wear over several hours.

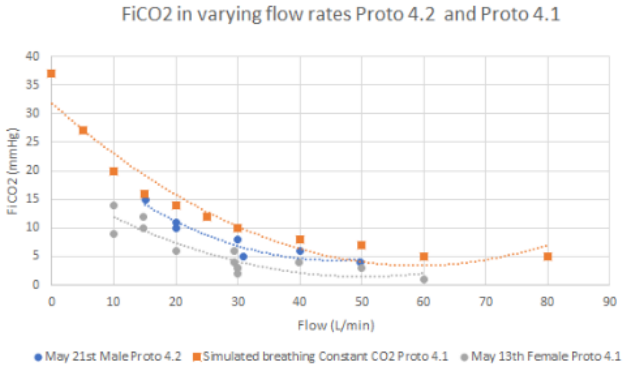

Fig. S1. As the inspiratory flow increases, $FiCO_2$ concentrations decrease. After about 30 L/min, acceptable $FiCO_2$ levels are achieved (n=3).

C. PEEP Range Test

The PEEP range test evaluated the performance of the CBH at different PEEP pressures. The pressure setting of the PEEP valve and the flow rate from the flow source was varied, while the pressure readings within the helmet were observed as seen in Figure S2.

The results for pressure containment were nearly identical between the CBH and the StarMed hood. The pressure inside the helmet was dependent on the flow rate in addition to the PEEP valve setting. This proved that the helmet can withstand high pressures of 30 cmH2O without observed failure of the components: hood, neck seal, and straps. It was further observed that the pressure in the hood is highly dependent on the flow at higher flow rates (>120 L/min).

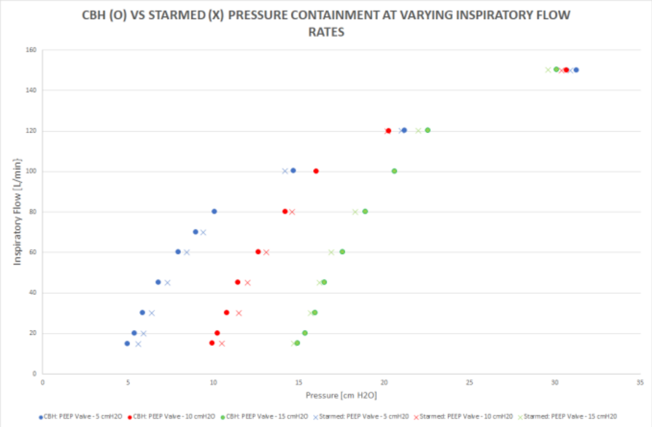

Fig. S2. As the inspiratory flow increases, pressure within the helmet increases as well. This graph shows that at higher inspiratory flow rates, pressure is highly dependant on the flow (n=2).

IV. Usability Testing

The usability test includes several factors that would be considered essential to our design and device effectiveness. Four (4) tests were conducted as part of the usability testing. Hood visibility - test subjects successfully performed tasks such as reading a book, working on a laptop, and using a mobile phone. Observers were able to see test subjects' facial expressions well. Ease and timeliness of donning and doffing of the CBH was tested, which is a vital factor in the event of an emergency. The COSMIC bubble helmet allows for quick donning by a single healthcare provider with an able and cooperative patient or two providers depending on the setting. Donning can be performed in less than 30 seconds and doffing by healthcare providers and/or patients takes less than 15 seconds. In the event of an emergency, the helmet's soft construction allows for emergency removal using a pair of scissors. The set-up and assembly test were used to assess the time required for healthcare providers to set up the helmet. With manual provided, the time required for a test subject to assemble the helmet was measured and observed. Based on our own results, device set-up time approximated five minutes. Operating noise of the helmet was also tested to ensure that the helmet was comfortable enough to be worn for extended periods of time, which may be required for NIV treatment. This test evaluated the comfort of wearing the helmet for up to 12 hours. Based on our results, operating noise did not exceed 87 decibels (dB). As per Usability Specification 5 our measured operating noise does not exceed that of the Canadian Federal Noise Regulation.

V. References

[1] P. Taccone, D Hess, P Caironi, and L. M. Bigatello. (2004, Sept.). Continuous Positive Airway Pressure Delivered with a 'Helmet': Effects on Carbon Dioxide Rebreathing. *Critical Care Medicine*. [Online abstract]. *32(10)*, pp. 2090-2096. Available: National Library of Medicine, https://pubmed.ncbi.nlm.nih.gov/. [Accessed June 7, 2020].

[2] N. Patroniti, G. Foti, A. Manfio, A Coppo, G. Bellani, and A. Pesenti. (2003, Aug.). Head Helmet versus Face Mask for Non-Invasive Continuous Positive Airway Pressure: a Physiological Study. *Intensive Care Medicine*. [Online abstract]. *29(10)*, pp. 1680-1687. Available: National Library of Medicine, https://pubmed.ncbi.nlm.nih.gov/. [Accessed June 6, 2020].

[3] R. Hernández-Molina, F. Fernández-Zacarías, I. Benavente-Fernández, G. Jiménez-Gómez, and S Lubián-López. (2017, Jan.). Head Helmet versus Face Mask for Non-Invasive Continuous Positive Airway Pressure: a Physiological Study. Noise and Health. [Online abstract]. 19(86), pp. 20-23. Available: https://pubmed.ncbi.nlm.nih.gov/28164935/. [Accessed June 12, 2020].

[4] M. Ferioli, *et al.* (2020, Mar.). Protecting healthcare workers from SARS-CoV-2 infection: practical indications. *European Respiratory Review*. [Online]. *29(155)*, p. 200068, Mar. 2020. Available: https://doi.org/10.1183/16000617.0068-2020. [Accessed June 7, 2020].
